# Ultra-Broadband Mid-Infrared Metamaterial Absorber Based on Multi-Sized Resonators

**DOI:** 10.3390/ma15155411

**Published:** 2022-08-05

**Authors:** Xiaojun Huang, Ziliang Zhou, Miao Cao, Rong Li, Cuizhen Sun, Xiaoyan Li

**Affiliations:** 1 College of Communication and Information Engineering, Xi’an University of Science and Technology, Xi’an 710054, China; 2 College of Physical Science and Technology, Northwestern Polytechnical University, Xi’an 710129, China

**Keywords:** metamaterial absorber, mid-infrared, multi-sized resonators, ultra-broadband

## Abstract

Mid-infrared metamaterial absorbers have many applications in the field of infrared detection, infrared thermal energy utilization, radiation refrigeration, invisible camouflage, etc. In this study, we designed an ultra-broadband mid-infrared metamaterial absorber based on multi-sized resonators. The structure of the absorber consisted of a gold substrate and nine resonators. The simulated results showed that the absorptivity of the absorber was higher than 90% in the 8.33–15.09 μm waveband with an average absorptivity of 95.17%. The energy distributions of the electric and magnetic fields were introduced to investigate the physics of broadband absorption. Moreover, we combined the multi-layer structure with the plane random arrangement structure to achieve a balance between thickness and width. Our study further illustrates the potential application of multi-sized resonators in metamaterial absorbers to realize high absorptivity and ultra-broadband to improve the performance of devices applied in infrared detection, radiation refrigeration, and other fields.

## 1. Introduction

As a composite with an artificial structure, metamaterials have unique properties that many natural materials do not have, such as a negative dielectric constant [1], negative permeability [2], negative refractive index [3], and epsilon-near-zero [4]; therefore, metamaterials research has become a popular area in recent years. Many fields are applying metamaterials to achieve specific characteristics, such as polarization control [5,6], electromagnetic (EM) stealth [7,8], radar antenna [9,10], photodetectors [11], tunable color filters [12], beam steering [13], etc. 

Since the first perfect metamaterial absorber (MA) was presented by Landy [14] in 2008, the EM metamaterial absorber research area has rapidly become popular, and many MAs have emerged. The absorption band of MAs has extended from the microwave band [15] to the terahertz [16,17,18,19], infrared [20,21,22], visible [23], and ultraviolet [24] bands. MAs have various features: tunable [25], cross-band absorption [26], polarization-insensitive [27,28,29], oblique-incidence [30,31,32,33], etc. Additionally, MAs apply multi-sized resonators [34,35,36] to realize ultra-broadband and high absorptivity in the mid-infrared range. Zheng Qin et al. proposed a broadband MA based on the lossy metal ring, in the 7.76–14 µm waveband, of which the absorptivity is over 90% and the average absorptivity is 93.8% [37]. Yu Zhou et al. presented a small-sized absorber with an absorptivity higher than 90% in the 8–14 µm waveband (covering the entire long wavelength infrared band) with an average absorptivity of 94.5% [38]. Biao Wu et al. presented an ultra-broadband metamaterial absorber, with an absorptivity is greater than 90% in the 939–3988 nm waveband [39]. Imre Ozbay et al. demonstrated an ultra-broadband metamaterial absorber with absorption above 0.9 in the waveband of 800–2390 nm by using the extraordinary optical response of bismuth (Bi) [40]. Although mid-infrared MAs have good performance, combining high absorptivity and broad bandwidth is still challenging.

In this paper, we present an ultra-broadband mid-infrared metamaterial absorber with nine resonators placed on a gold substrate with the resonators arranged as a 3 × 3 five-layer unit cell. The absorptivity of this MA is greater than 90% in the 8.33–15.09 μm waveband with an average absorptivity of 95.17%. Our study illustrates the potential application of multi-sized resonators in metamaterial absorbers to realize higher absorptivity and ultra-broadband. The proposed MA has potential application value in the fields of infrared detection, infrared thermal energy utilization, radiation refrigeration, invisible camouflage, etc.

## 2. Design and Simulation

**Figure 1:**
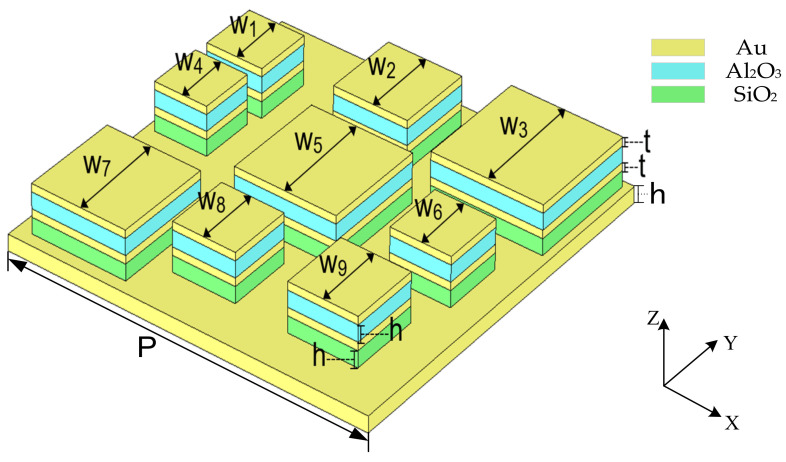
Schematic view of the Au, Al_2_O_3_, Au, SiO_2_, and Au 3 × 3 five-layer unit cell.

In Figure 1, we display the structure designed for the MA, which comprises nine square resonators of different sizes, consisting of four layers of Au, Al_2_O_3_, Au, and SiO_2_ placed on a square gold substrate, and we use the subscript w to indicate the sequence number of the resonators. Moreover, without considering the z-direction, we set the geometric center of the substrate as the coordinate origin; thus, the coordinates of resonator 1–9 center are (−4.7, 3.1), (−0.5, 4.0), (3.9, 3.9), (−4.8, 0.7), (0, 0), (4.1, −0.2), (−3.9, −4.0), (−0.5, 3.5), and (3.5, −3.5), respectively. The characteristics of the materials applied in the MA were the relative permittivity and loss tangent of Al_2_O_3_ and SiO_2_, and the conductivity of gold; the values of which are 2.28; 0.04, 3.9, and 0.025; and 4.56 × 10^7^ S/m, respectively. Additionally, we list the dimensions of the MA structural parameters in Table 1.

We carried out all the simulations using CST microwave studio 2020 software. We chose the frequency domain analyzer as the simulation solver to obtain the absorptivity of the MA with the simulation and calculation. The mesh type was tetrahedral. Further, in the x and y directions, we set the conditions of the boundary as the unit cell. Set as open add space, the condition of the boundary was applied in the direction of Z_max_, while the boundary condition in the Z_min_ direction was set as grounding (E_t_ = 0).

## 3. Results and Discussion

To calculate the absorptivity of the proposed MA, we used the equation $A=1-R-T=1-\left|S_{11}{|}^{2}-\right|S_{21}{|}^{2}$. In this equation, R and T represent the reflection ratio and transmission ratio, respectively, so that $S_{11}$ and $S_{21}$ represent the coefficient of reflection and transmission, respectively. In the range of mid-infrared, the gold substrate with thickness much thicker than skin depth caused the value of the transmission ratio for MA to be equal to zero. Therefore, the above equation can be further expressed as $A=1-R=1-|S_{11}{|}^{2}$.

**Figure 2:**
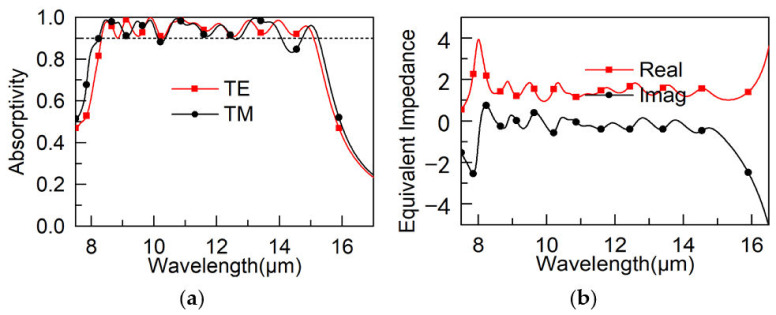
(**a**) Absorptivity of the MA; (**b**) real and imaginary parts of equivalent impedance of the MA.

The absorptivity of the MA is shown in Figure 2. In Figure 2a, the absorptivity under the transverse electric (TE) mode is greater than 0.9 in the 8.33–15.09 μm, and at the resonant peak of 9.88 μm, the absorptivity is over 99.9%. The performance of absorptivity under the Transverse Magnetic (TM) mode was not as good as under the TE mode due to the asymmetrical structure of the MA. Perfect absorption can be obtained when the equivalent impedance of MA and free space match. Hence, the performance of the proposed MA is explained by the equivalent impedance of the MA displayed in Figure 2b. As shown, at the resonant peaks of 8.5, 9.13, 9.88, 10.86, 11.26, 12.07, 13.03, 13.96, and 14.84 μm, the real and imaginary parts of the equivalent impedance for the proposed MA were nearly one and zero, respectively. Moreover, the equivalent impedance of the real and imaginary parts had a value around one and zero in the whole range of 8.33–15.09 μm, respectively. In summary, the proposed MA demonstrated perfect absorption because the equivalent impedance of the MA and corresponding value of the free space matched. 

**Figure 3:**
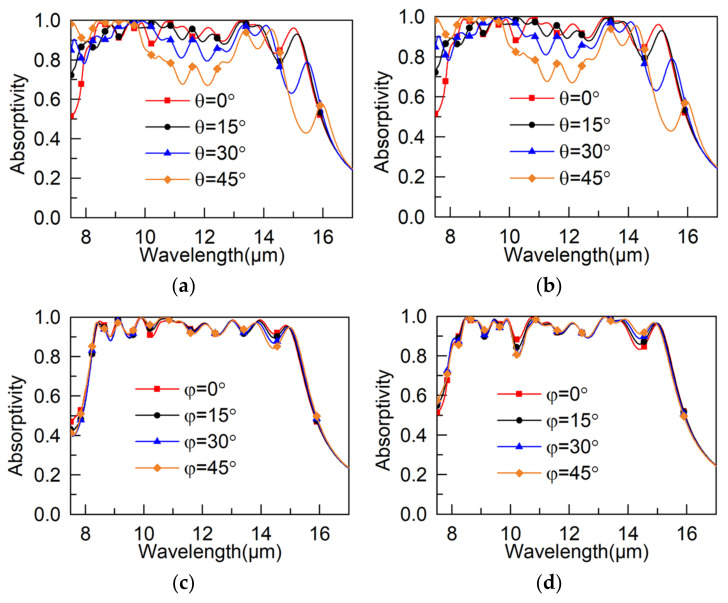
Absorptivity with (**a**) oblique incident of TE wave, (**b**) oblique incident of TM wave, (**c**) different polarization angles of TE wave, and (**d**) different polarization angles of TM wave.

Investigating how the incidence and polarization affect the absorptivity, we further illustrated the performance of the proposed MA, and the results are displayed in Figure 3. In Figure 3a,b, compared with the results of a normal incident, we can see that under both the TE and TM modes, the performance of the MA was significantly worse with increasing incident angle because the resonance conditions can be changed with the obliquity of the incident wave. Meanwhile, Figure 3c,d show that the absorptivity changed across the whole operating frequency band with increasing polarization angle, which was a result of the non-central rotational symmetry structure of the MA.

**Figure 4:**
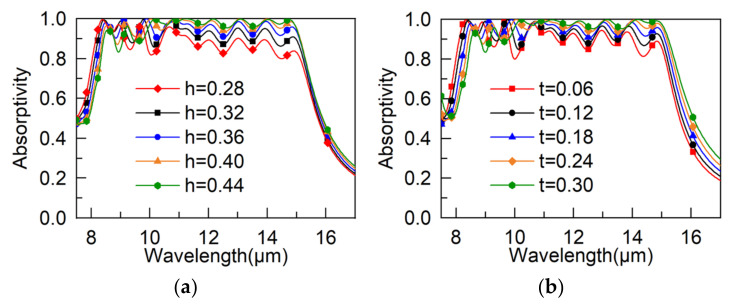

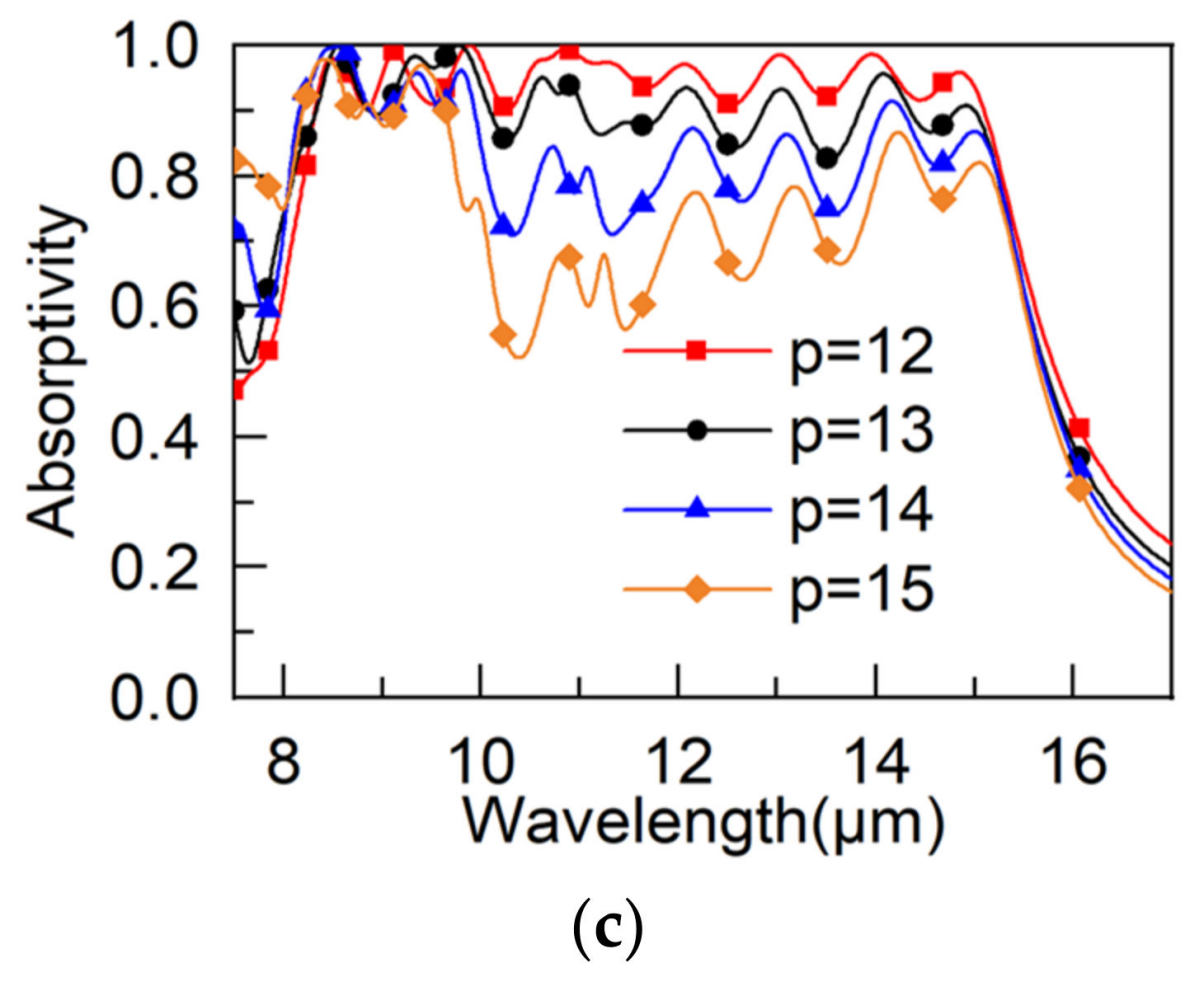
Absorptivity with different (**a**) thickness of dielectric layers, (**b**) thickness of gold layers, and (**c**) period geometry.

We further simulated how the changes in the structure parameters affect the absorptivity, and the result is shown in Figure 4. In Figure 4a, we can see that the absorptivity is rising with increasing h when the wavelength is longer than 10.6 μm, and in the 8.33–10.6 μm range, the absorptivity of h = 0.36 μm shows the widest bandwidth. Figure 4b shows the relationship between absorptivity and the structure parameter t; when the wavelength is over 10.17 μm, there is a positive correlation between absorptivity and the value of t, while in the 8.33–10.17 μm range, the relationship is the opposite. Figure 4c shows that the structure period P has an obvious influence on the absorptivity. The performance of the MA deteriorates as the value of *p* becomes larger, and the minimum value of *p* is 12 μm due to the structure of the MA.

**Figure 5:**
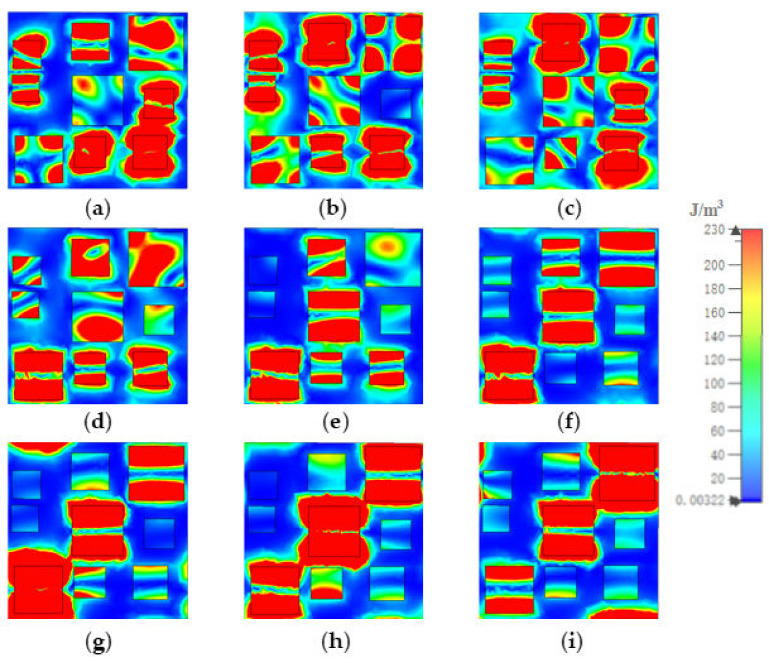
The distribution of electric field energy in gold substrate at (**a**) 8.5, (**b**) 9.13, (**c**) 9.88, (**d**) 10.86, (**e**) 11.26, (**f**) 12.07, (**g**) 13.03, (**h**) 13.96, and (**i**) 14.84 μm.

**Figure 6:**
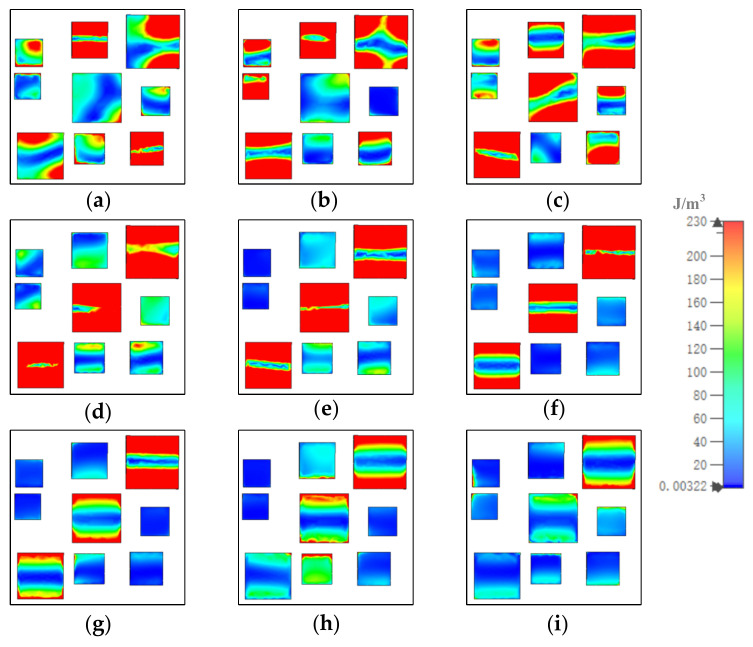
The distribution of electric field energy in middle gold layer at (**a**) 8.5, (**b**) 9.13, (**c**) 9.88, (**d**) 10.86, (**e**) 11.26, (**f**) 12.07, (**g**) 13.03, (**h**) 13.96, and (**i**) 14.84 μm.

**Figure 7:**
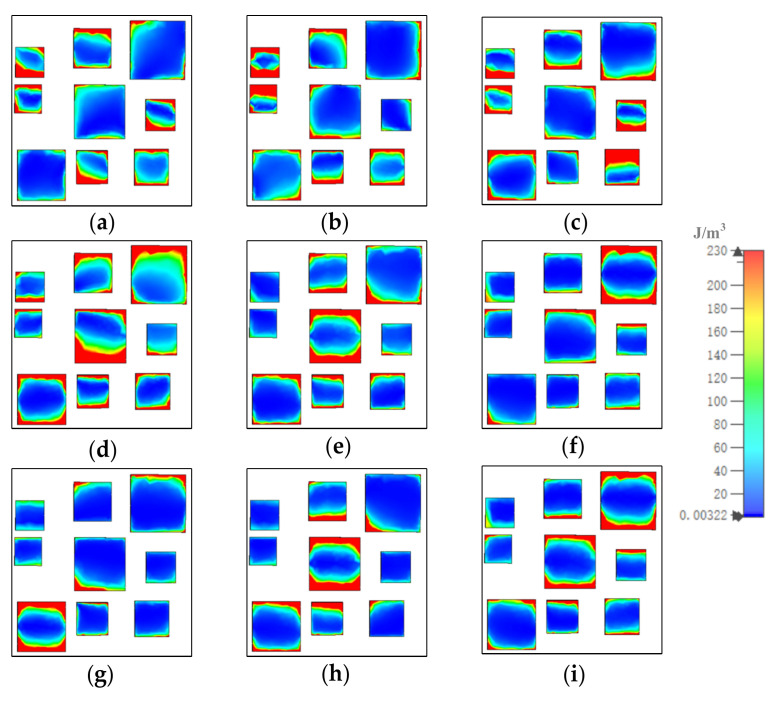
The distribution of electric field energy in top gold layer at (**a**) 8.5, (**b**) 9.13, (**c**) 9.88, (**d**) 10.86, (**e**) 11.26, (**f**) 12.07, (**g**) 13.03, (**h**) 13.96, and (**i**) 14.84 μm.

To investigate the distribution of the electric and magnetic fields, we analyzed the absorption mechanism of the MA. Figure 5, Figure 6, and Figure 7 show how the electric field energy distributed in each of the gold layers. At 8.5 μm, in gold substrate, most of the electric field energy distributed in the up and down sides under resonators 1, 2, 3, and 4; under resonator 7, the electric field energy distributed in four corners; and under resonators 6, 8, and 9, the electric field energy was almost fully covered. The distribution out of the range illustrated the coupling of resonators. In the middle gold layer, the electric field energy mainly distributed in the up and down sides of resonators 2, 3, and 9, and in several corners of resonators 1, 5, 6, 7, and 8. In the top gold layer, the electric field energy mainly distributed in the up and down sides of resonators 1, 2, 4, 6, 8, and 9. At other resonator points, the electric field energy distributions were similar to those at 8.5 μm, although the number and position of the resonator in which the most field energy distributes were different.

**Figure 8:**
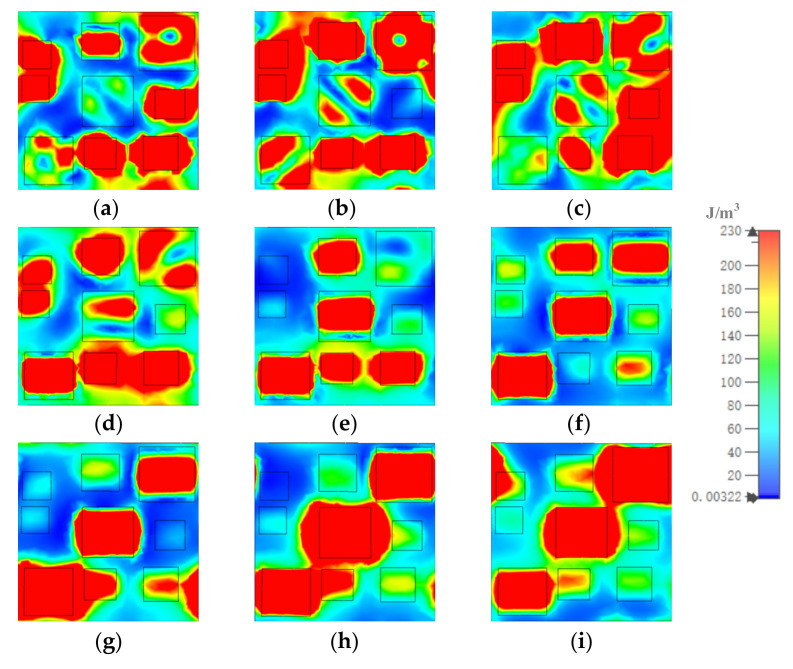
The distribution of magnetic field energy in gold substrate at (**a**) 8.5, (**b**) 9.13, (**c**) 9.88, (**d**) 10.86, (**e**) 11.26, (**f**) 12.07, (**g**) 13.03, (**h**) 13.96, and (**i**) 14.84 μm.

**Figure 9:**
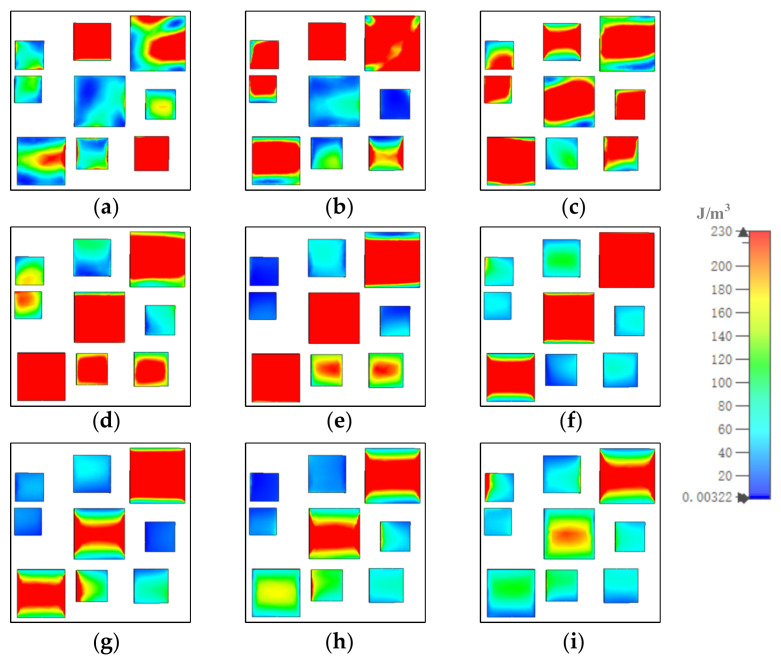
The distribution of magnetic field energy in middle gold layer at (**a**) 8.5, (**b**) 9.13, (**c**) 9.88, (**d**) 10.86, (**e**) 11.26, (**f**) 12.07, (**g**) 13.03, (**h**) 13.96, and (**i**) 14.84 μm.

**Figure 10:**
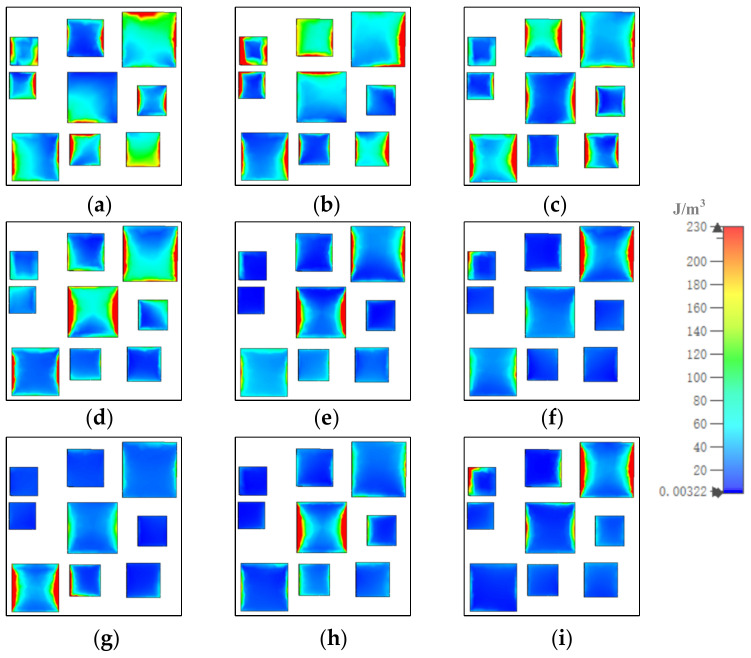
The distribution of magnetic field energy in top gold layer at (**a**) 8.5, (**b**) 9.13, (**c**) 9.88, (**d**) 10.86, (**e**) 11.26, (**f**) 12.07, (**g**) 13.03, (**h**) 13.96, and (**i**) 14.84 μm.

Figure 8, Figure 9 and Figure 10 show how the magnetic field energy distributed in each of the gold layers. At 8.5 μm, displayed in Figure 8, Figure 9 and Figure 10a, in gold substrate, most of the magnetic field energy distributed under resonators 1, 2, 3, 4, 6, 8, and 9. As shown, the magnetic field energy almost covered the whole space under resonators 1, 4, 6, 8, and 9. The distribution out of the range illustrated the coupling of resonators. In the middle gold layer, most of the magnetic energy distributed in the whole range of resonators 2 and 9, and large amounts of magnetic energy distributed in a horizontal stripe in the middle of resonators 3 and 7. In the top gold layer, the magnetic field energy mainly distributed in the left and right sides of resonators 1, 2, 3, 4, 6, 7, and 9. At other resonator points, the magnetic field energy distributions were similar to those at 8.5 μm, although the number and position of the resonator in which the most field energy distributes were different. 

We concluded that most of the energy distributed in the gold substrate and middle gold layer, and the remaining minor electric field energy and magnetic field energy distributed in the up and down, and left and right sides of the resonator in the top gold layer, respectively. Not only the resonators but also the coupling of several resonators with different sizes contributed to the generation of the resonant peaks. Additionally, we present a comparison between the proposed MA and those reported in several previous works in Table 2, which illustrates the high performance of the proposed MA.

## 4. Conclusions

In summary, we proposed a metamaterial absorber characterized by ultra-broadband absorption in the mid-infrared range based on multi-sized resonators. Initially, we designed a 3 × 3 five-layer structure with Au, Al_2_O_3_, and SiO_2_ to absorb mid-infrared waves. Then, we changed the size of each resonator to expand the bandwidth because resonators with different sizes have different operation wavelengths and the relationship between them is positive. We further rearranged the resonators to change their distances, which created variation in the coupling condition between the resonators. Therefore, the performance of our MA was improved. By conducting simulations and calculations, we ensured the final design of the proposed MA. The results showed that the absorptivity of the proposed MA in the 8.33–15.09 μm waveband was higher than 90%, with an average absorptivity of 95.17%, and the absorption bandwidth was about 6.76 μm. The proposed MA has promising application value in the fields of infrared detection, infrared thermal energy utilization, radiation refrigeration, invisible camouflage, etc. 

## Figures and Tables

**Table 1:** Dimension of the MA structural parameters.

Parameter	Size (μm)	Parameter	Size (μm)
P	12	w_4_	1.8
t	0.18	w_5_	3.4
h	0.36	w_6_	2.0
w_1_	1.9	w_7_	3.2
w_2_	2.5	w_8_	2.1
w_3_	3.7	w_9_	2.3

**Table 2:** Comparison between proposed MA and those designed in previous works.

Reference	Operation Bandwidth	Relative Bandwidth	Average Absorptivity
[37]	7.76–14 μm	57.35%	93.8%
[38]	8–14 μm	54.54%	94.5%
[41]	8–12 μm	40%	92.1%
[42]	7.5–13.25 μm	55.42%	91.7%
Proposed MA	8.33–15.09 μm	57.73%	95.17%

## Data Availability

The data presented in this study are available on request from the corresponding author.
